# Long-Term Stabilization of Two-Dimensional Perovskites by Encapsulation with Hexagonal Boron Nitride

**DOI:** 10.3390/nano9081120

**Published:** 2019-08-03

**Authors:** Michael Seitz, Patricia Gant, Andres Castellanos-Gomez, Ferry Prins

**Affiliations:** 1 Condensed Matter Physics Center (IFIMAC), Autonomous University of Madrid, 28049 Madrid, Spain; 2 Materials Science Factory, Instituto de Ciencia de Materiales de Madrid, Consejo Superior de Investigaciones Científicas, 28049 Madrid, Spain

**Keywords:** perovskites, stability, exfoliation, encapsulation, two-dimensional materials, Ruddlesden-Popper

## Abstract

Metal halide perovskites are known to suffer from rapid degradation, limiting their direct applicability. Here, the degradation of phenethylammonium lead iodide (PEA_2_PbI_4_) two-dimensional perovskites under ambient conditions was studied using fluorescence, absorbance, and fluorescence lifetime measurements. It was demonstrated that the long-term stability of two-dimensional perovskites could be achieved through the encapsulation with hexagonal boron nitride. While un-encapsulated perovskite flakes degraded within hours, the encapsulated perovskites were stable for at least three months. In addition, encapsulation considerably improved the stability under laser irradiation. The environmental stability, combined with the improved durability under illumination, is a critical ingredient for thorough spectroscopic studies of the intrinsic optoelectronic properties of this material platform.

## 1. Introduction

Metal halide perovskites (APbX_3_, A = methylammonium, formamidinium, Cs; X = I, Br, Cl) have recently emerged as a promising material class for light-harvesting [1,2,3,4] and light-emitting [5,6,7,8,9,10,11] applications. Most prominently, perovskite solar cells have reached light collection efficiencies of well over 20% just a few years after their first reports [4]. Such rapid advances have been made possible through an excellent combination of favorable properties, such as solution processability at low temperatures [2,12], remarkable tolerance to defects [13,14,15,16,17], high absorption coefficients [2,12], long-range ambipolar charge transport characteristics [18,19,20], and the broad tunability of their bandgap, which is freely adjustable from the ultra-violet into the infra-red [21,22,23].

Despite these advantages, the study and applicability of metal halide perovskites remain challenging due to their poor environmental stability [3,24,25,26]. Perovskites normally degrade within several hours or days under the ambient condition with oxygen, moisture, light irradiation, and heat playing an important role in the degradation process [3,24,25,26]. Findings of several studies on lead iodide perovskites suggest that during degradation, the bulk perovskite CH_3_NH_3_PbI_3_ decomposes into the volatile species I_2_ and CH_3_NH_3_ (methylammonium), leaving behind PbI_2_ [27,28,29,30]. The cause of this degradation is ascribed to oxygen [29,31,32], moisture [27,28,29,33,34], photoactivation [27,32,35,36], and/or heat [3,24,25,29,35].

One possible route to improve the stability of perovskites is through tuning of their chemical composition, for example, by exchanging the methylammonium cation with the less volatile formamidinium [37], cesium [38], rubidium [39], or a mixture thereof [40,41,42,43]. Also, a partial or complete substitution of iodine with bromine [44,45], chlorine [46], or a mixture of the two [47] has been shown to yield more stable perovskites [26]. However, despite these efforts, the stability of the perovskite crystals remains limited. Another promising strategy to significantly improve the stability of perovskite devices is to reduce the dimensionality of the perovskite crystals [26]. A prominent example is a class of layered two-dimensional (2D) perovskites, which have higher formation energy than their bulk counterparts due to closely bound ligands, which separate the inorganic layers. Notably, Grancini et al. [48] have shown that 2D/3D perovskite solar modules show prolonged stability.

An alternative strategy to improve the stability of perovskites is through encapsulation, which prevents water and air molecules from reaching the perovskite crystal in the first place [24,25,49,50,51,52,53]. For example, by encapsulation of a complete photovoltaic device, Gevorgyan et al. [54] produced a perovskite solar cell, which was stable for over a year. Furthermore, it has been shown that 2D materials suffering from air-induced degradation, such as black phosphorus, can be protected by encapsulation with hexagonal boron nitride (hBN) [55,56]. Indeed, recent studies have shown that this hBN encapsulation on such a smaller level is also possible for perovskites, providing improved resistance to heat, moisture, and light irradiation [30,57,58,59]. These studies have reported the stability of encapsulated perovskites for up to several hours. A long-term stabilization, however, is yet to be demonstrated.

Here, we studied the feasibility of the hBN encapsulation strategy to provide long-term stability of phenethylammonium lead iodide (PEA_2_PbI_4_) 2D perovskites. Specifically, we employed a double-sided encapsulation (hBN/perovskite/hBN) in which the perovskite flake was fully sealed by hBN. While unprotected flakes degraded within hours, the encapsulated perovskite flakes displayed no observable degradation in their optical properties for at least three months. Further, we report a significantly improved resistance towards laser radiation. The environmental stability, combined with the improved durability under illumination, is a critical ingredient for thorough spectroscopic studies of the intrinsic optoelectronic properties of this material platform. This is of particular importance for thin flakes, where degradation effects are more pronounced.

## 2. Materials and Methods

### 2.1. Chemicals

Chemicals were purchased from commercial suppliers and used as received: lead (II) iodide PbI_2_ (Sigma Aldrich, St. Louis, MO, USA; 900168-5G), phenethylammonium iodide (PEAI, Sigma Aldrich, St. Louis, MO, USA; 805904-25G), γ-butyrolactone (Sigma Aldrich, St. Louis, MO, USA; B103608-500G), hexagonal boron nitride hBN (HQ Graphene, Groningen, Netherlands).

### 2.2. 2D Perovskite Synthesis

Layered perovskites were synthesized under ambient laboratory conditions following the over-saturation techniques reported previously [60,61]. In a nutshell, the PbI_2_ (200 mg) and PEAI (216 mg) were mixed in a 1:2 molar ratio and dissolved in γ-butyrolactone (300 µL). The solution was heated to 70 °C, and more γ-butyrolactone (0–200 µL) was added until all the precursors were completely dissolved. The vial was kept open, allowing the solvent to slowly evaporate. After 2–3 days, millimeter-sized crystals formed in the solution, which was subsequently cooled down to room temperature. For this study, we drop cast some of the remaining supersaturated solution on a glass slide (the millimeter-sized crystals were used for another study), heated it to 70 °C with a hotplate, and after the solvent was evaporated, PEA_2_PbI_4_ crystals with crystal sizes of up to several hundred microns were formed. The saturated solution could be stored and re-used to produce freshly grown 2D perovskites within several minutes.

### 2.3. Exfoliation

The synthesized PEA_2_PbI_4_ perovskite crystals were mechanically exfoliated using the Scotch tape method (Nitto, Osaka, Japan; SPV 224). After an appropriate flake thickness was obtained, the flakes were exfoliated one last time with polydimethylsiloxane (PDMS, Gelfilm from Gelpak^®^, Hayward, CA, USA), which is better suited for the precise placement of the flakes. Following this method, we were able to obtain thin single crystals with sizes up to hundreds of microns. After several exfoliations between two scotch tapes and exfoliation with the PDMS, we transferred the flakes on a glass substrate. We found that retracting the tape and PDMS stamp quickly transferred thick and large crystals, while slow retraction yielded the thinner crystals.

### 2.4. Characterization

Fluorescence and absorption spectra were recorded using a spectrograph and an EMCCD camera (Princeton Instruments, Trenton, NJ, USA; SpectraPro HRS-300, ProEM HS 1024BX3) with a 300 g/mm grating with a blaze of 500 nm. The samples were excited by a LED (Thorlabs, Newton, NH, USA; M385PLP1-C5, λ = 385 nm) for fluorescence measurements (0.55 mWcm^−2^, 1 min exposure) and by a white light LED (Thorlabs, Newton, NH, USA; MCWHLP1) for absorbance measurements (0.12 mWcm^−2^, 1 min exposure). Fluorescence lifetime measurements were performed with a laser diode of λ = 405 nm (PicoQuant, Berlin, Germany; LDH-D-C-405, PDL 800-D, Pico-Harp 300) and an avalanche photodiode (APD, Micro Photon Devices PDM, Bolzano, Italy). The repetition rate was 1 MHz, and the peak fluence per pulse was 1 nJcm^−2^. Samples were stored in the dark and under ambient conditions (20 °C, 35% relative humidity) in between measurements. X-ray diffraction (XRD) was performed using a D8 Advance (Bruker, Billerica, MA, USA) operating at 40 kV and 30 mA using a copper radiation source (1.54060 Å). XRD measurements were taken from the drop cast perovskite films.

## 3. Results and Discussion

**Figure 1:**
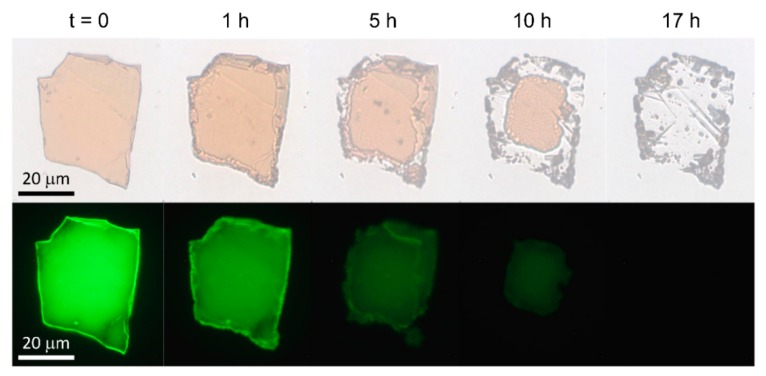
Complete degradation of an exfoliated phenethylammonium lead iodide (PEA_2_PbI_4_) two-dimensional (2D) perovskite flake within 17 h of ambient conditions. First- and second-row show transmission and fluorescence micrographs of the flake, respectively.

Two-dimensional perovskites PEA_2_PbI_4_ were synthesized by a one-step drop casting from a saturated precursor solution. The crystalline integrity of the perovskite film was confirmed by performing x-ray diffraction (XRD) analysis. As shown in Figure S1, the XRD measurement revealed evenly spaced peaks, which confirmed the *n* = 1 perovskite structure with a spacing of 1.63 nm between the inorganic layers, consistent with previously reported values [62,63,64]. Through mechanical exfoliation of the crystalline perovskite film, we obtained 10–200 µm large single-crystalline multilayer 2D flakes, as shown in Figure 1 [65]. Fluorescence and absorbance spectroscopy on the flakes revealed the characteristic green fluorescence at 527 nm known for PEA_2_PbI_4_ (Figure S2).

Only minutes after their exfoliation, the 2D perovskite flakes started to degrade into transparent and non-emissive PbI_2_ [27,28,29,30]. The degradation started at the surface and progressed into the flake, as can be seen from the transmission and fluorescence images in Figure 1. The lateral degradation of the flake was faster. We assign this to the easier diffusion of water, oxygen, and reaction products along the inorganic layers as compared to the layer-by-layer diffusion through the inorganic layers. However, the decrease of fluorescence and absorbance in the center of the flake, well before the lateral degradation reached the center of the flake, suggests that layer-by-layer degradation was also present, although slower than the lateral degradation along the inorganic layers. It is important to notice that in thin films, the lateral degradation is hindered through neighboring crystallites, making it harder for air molecules to penetrate the perovskite film and thereby slowing down the overall degradation process. As a result, the degradation time of perovskites might vary for different studies, since the speed of the degradation is thickness and lateral-size dependent.

**Figure 2:**
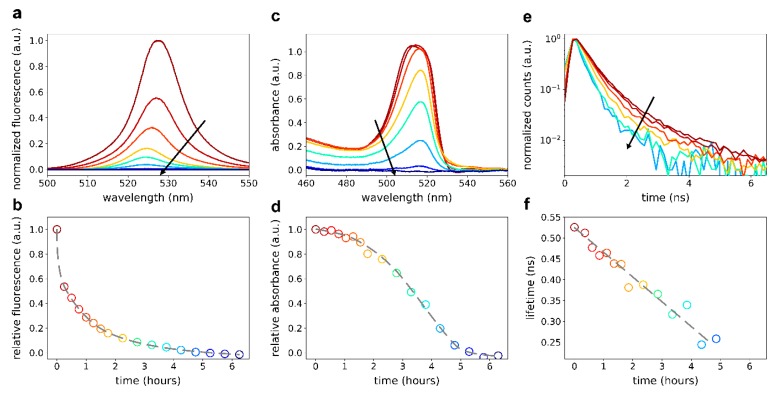
Spectral properties of a PEA_2_PbI_4_ 2D perovskite flake for different times under ambient exposure. (**a**,**c**,**e**) Fluorescence, absorbance, and fluorescence lifetime traces, respectively, for different times of ambient exposure. Traces shown correspond to times 0, 0.25, 1, 2.25, 3.25, 4.25, 5.25, and 6.25 h. Fluorescence lifetime traces are only shown for t < 5 h as the data was too noisy for later times. (**b**) Total fluorescence intensity from (**a**) normalized to the measured intensity at t = 0. (**d**) Integrated absorbance (455–594 nm) normalized to the measured absorbance at t = 0. (**f**) Extracted 1/e lifetime from the fluorescence lifetime traces in (**e**). Gray lines in (**b**), (**d**), (**f**) are guides to the eye.

To study the time-dependent degradation of 2D perovskite flakes, we monitored the fluorescence, absorbance, and fluorescence lifetime of the flakes in more detail (Figure 2). During the measurements, exposure to light was minimized to avoid light-induced degradation as much as possible. As can be seen in Figure 2b, the degradation was most prominently visible in the fluorescence measurements. After a first initial drop, which we attribute to the formation of non-radiative trap states at the surface, a slower decay of fluorescence intensity (between 15 min and 5 h) was observed. This could be explained by a diffusion-limited progression of degradation into the perovskite flake, during which the fluorescence dropped exponentially (Figure S3). In addition to the decreasing fluorescence intensity, we observed a 4 nm blueshift of the emission peak. Likewise, we observed a decrease in the first absorption peak (Figure 2c,d) and the fluorescence lifetime (Figure 2e,f) although less rapidly than for the fluorescence. The faster degradation of the fluorescence as compared to the absorptive properties and the simultaneous decrease in fluorescence lifetime suggested the formation of additional non-radiative decay channels. We would like to note that the smaller size of the flake of Figure 2 lead to a faster complete degradation than that of the larger flake depicted in Figure 1.

**Figure 3:**
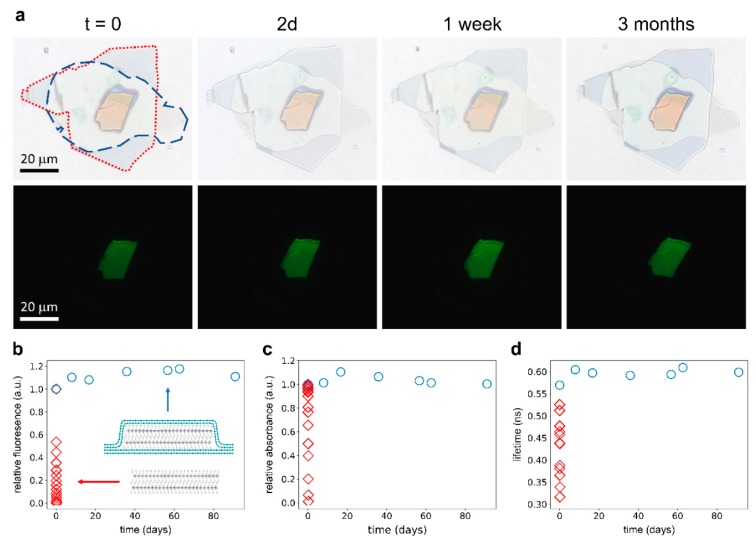
Spectral properties of a PEA_2_PbI_4_ 2D perovskite flake, which was encapsulated between two hexagonal boron nitride (hBN) layers; (**a**) Transmittance (top) and fluorescence (bottom) micrographs of the encapsulated perovskite flake showing no sign of degradation after exposure to ambient conditions for over three months. In the top left micrograph, we highlight the edges of the bottom (blue dashed) and top (red dotted) hBN flake; (**b**) Total fluorescence intensity of an encapsulated (circles) and un-encapsulated (diamonds) perovskite flake for different times under ambient condition. The intensities were normalized to the measured value at *t* = 0; (**c**) Integrated absorbance (455–594 nm) of an encapsulated (circles) and un-encapsulated (diamonds) perovskite flake for different times under ambient condition. The intensities were normalized to the measured value at *t* = 0; (**d**) 1/e lifetime of an encapsulated (circles) and un-encapsulated (diamonds) perovskite flake for different times under ambient condition.

To prevent rapid degradation, we fully encapsulated perovskite flakes between two hBN layers. First, an hBN flake was transferred onto a glass slide using mechanical exfoliation with a stamp of PDMS. In the next step, a perovskite flake, obtained by the same method, was placed onto the first hBN flake using an all dry deterministic placement method and finally the perovskite was encapsulated with a second mechanically exfoliated hBN flake (exposure of the perovskite flake to ambient conditions is <10 min), using the same deterministic placement technique [66]. We chose double-sided hBN encapsulation since the hBN/hBN interface is known to yield a more air-tight seal as compared to a single-sided encapsulation where the seal is formed by an hBN/SiO_2_ interface [55,58,67]. In the latter case, air can slowly pass through the rough and porous SiO_2_. In Figure 3a, we show a transmission microscopy image of the hBN/perovskite/hBN stack with dotted lines identifying the different layers.

Following the same procedures as for the un-encapsulated perovskite flake, we monitored the fluorescence, absorbance, and fluorescence lifetime of the encapsulated flake. After three months, no significant degradation was observed on the encapsulated flake. This can be seen qualitatively in Figure 3a–d, where we show that an encapsulated flake maintains its optical properties for three months, while an un-encapsulated flake degrades within 5 h. For the encapsulated flake, the fluorescence and the fluorescence lifetime even increased slightly. We attributed this increase in fluorescence to photobrightening through a photo-induced halide redistribution that occurred during our spectral measurements, despite the low fluences during our measurements [68]. The fluorescence, absorbance, and fluorescence lifetime traces of the encapsulated flake are shown in Figure S4. Figure S5 shows a partially encapsulated flake with a small leak in the hBN seal. It is visible that the degradation started at the leak and slowly progressed into the flake. However, the degradation was significantly slowed down because the diffusion of the air molecules was limited by the small hole in the hBN seal. Even after one month, the center of the flake was not yet significantly affected by degradation.

**Figure 4:**
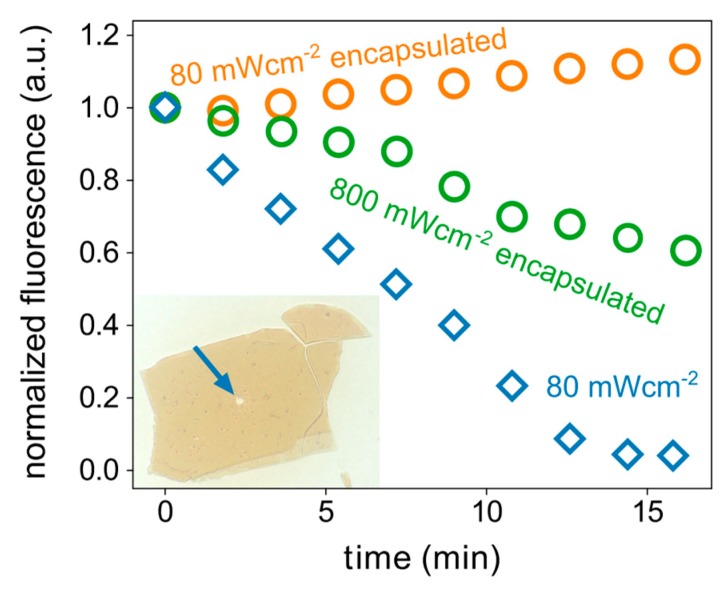
Degradation of PEA_2_PbI_4_ 2D perovskite flakes under 405 nm laser illumination. The graph shows the normalized (to *t* = 0) fluorescence intensity of encapsulated (circles) and un-encapsulated (diamonds) perovskite flakes for different times of laser exposure. The un-encapsulated flake degraded rapidly within only 15 min under 80 mWcm^−2^ of laser light. For the same laser intensity, the encapsulated sample did not show any effect of degradation. When increasing the intensity tenfold to 800 mWcm^−2^, also the encapsulated sample started to degrade and decreased to around 60% of its initial brightness after 15 min of exposure. The inset shows an un-encapsulated flake (~70 µm in width) after 15 min of exposure to 80 mWcm^−2^ of laser light. The flake degraded locally and became transparent, where the laser hit the flake (indicated by the arrow).

To test the stability of an hBN encapsulated perovskites under light illumination, we monitored the time-dependent fluorescence intensity during exposure to laser light. We used a Picoquant 405 nm laser, which was focused down on the perovskite flake, creating a laser spot with a full-width half max of around 1 µm. Using a laser intensity of 80 mWcm^−2^, the un-encapsulated perovskite flake completely degraded within only 15 min, as shown in Figure 4, with a transmission photograph of the un-encapsulated flake after exposure to laser light. The local degradation through the laser is nicely visible as the degraded region (indicated with a blue arrow) does not absorb any visible light. On the other hand, the encapsulated flake showed no sign of degradation. In contrast, the fluorescence intensity even slightly increased, which could be due to the photobrightening effect [68]. It was necessary to increase the laser intensity ten times to observe a sizeable drop in the fluorescence in the encapsulated flake, and it was still less pronounced than the drop observed in the un-encapsulated flake with ten times lower laser intensity.

## 4. Conclusions

In conclusion, we have shown that it is possible to prevent the degradation under ambient conditions of PEA_2_PbI_4_ perovskite flakes through double-sided encapsulation with hBN and that hBN encapsulated flakes are stable for at least three months. Further, encapsulated flakes show increased resistance to laser illumination. As a result, hBN encapsulation allows more thorough spectroscopic studies of the intrinsic optoelectronic properties of this material platform. Especially, studies of few-layer perovskites can benefit from increased stability, as they are particularly sensitive to environmental and light exposure. The presented encapsulation technique is not restricted to hBN but can be extended to other 2D materials that prevent the diffusion of water and air molecules, offering the possibility to have an integrated encapsulation in perovskite-2D material heterostructures.

## Supplementary Materials

The following are available online at https://www.mdpi.com/2079-4991/9/8/1120/s1, Figure S1: XRD data, Figure S2: Fluorescence and absorbance of PEA_2_PbI_4_, Figure S3: Logarithmic plot of data from Figure 2b, Figure S4: Fluorescence, absorbance, and lifetime traces of the encapsulated flake, Figure S5: Fluorescence and transmittance images of an un-encapsulated and partially encapsulated flake.
