# Supplementary material for: Long-Term Stabilization of Two-Dimensional Perovskites by Encapsulation with Hexagonal Boron Nitride
Source: Nanomaterials (Basel). 2019 Aug 3;9(8):1120. doi: 10.3390/nano9081120 (PMC6724044; doi:10.3390/nano9081120)
Supplement: Supplementary file 1 [file nanomaterials-09-01120-s001.pdf]

nanomaterials

MDPI

SUPPORTING INFORMATION

Figure S1a shows the x-ray diffraction (XRD) of a freshly dropcast film of PEA_2PbI_4 perovskite. The XRD data reveals evenly spaced peaks of 5.5° (2θ). Using Bragg's law

$$n\lambda = 2d\sin(\theta) \rightarrow \sin(\theta) = \frac{\lambda}{2d}n \qquad (1)$$

and a linear fit through the peak positions (Figure S1b), one can extract a spacing of 1.63 nm of the inorganic layers (λ = 1.54060 Å). Additionally, the absence of other diffraction peaks from the perovskite unit cell shows that the inorganic layers of the 2D perovskite grew parallel to the substrate.

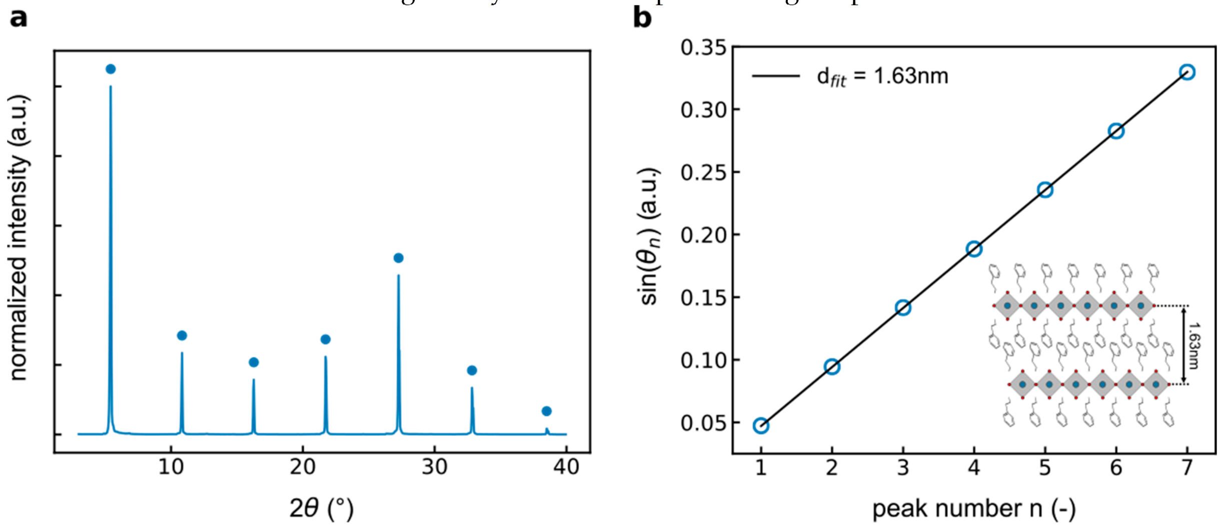

Figure S1. a) X-ray diffraction of PEA2PbI4 with evenly spaced peaks. b) A linear fit through the peak positions and using Bragg's law reveals a spacing of 1.63 nm of the inorganic layers. Inset shows the sketch of a 2D perovskite structure, indicating the measured spacing of the inorganic layers.

Figure S2 shows a characteristic fluorescence and absorbance spectra of an exfoliated PEA2PbI4 flake.

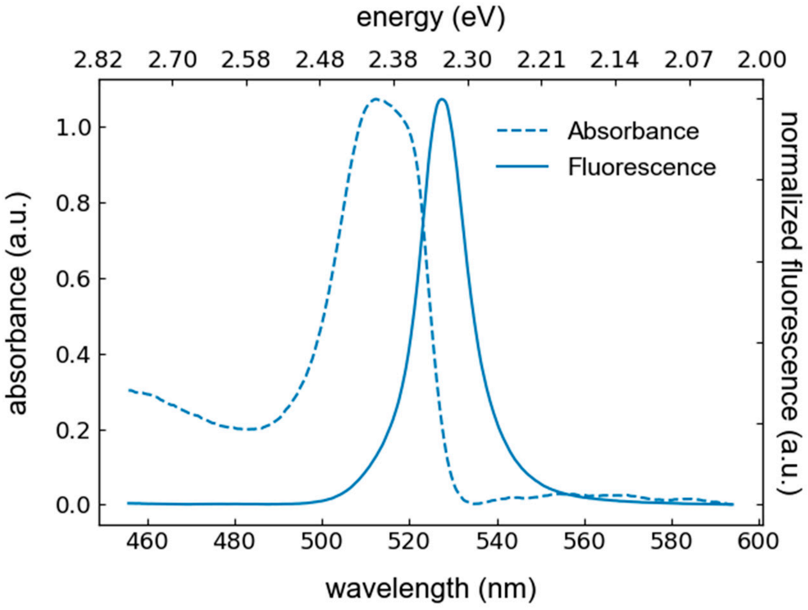

Figure S2. Fluorescence (solid) and absorbance (dashed) spectra of an exfoliated PEA_2PbI_4 2D perovskite flake.

Figure S3 shows the total normalized (to t = 0) fluorescence intensity of the un-encapsulated perovskite flake from Figure 2 of the main text. The logarithmic scale depicts three different stages. First a rapid drop to around 50% of emission intensity, which we attribute to the formation of non-radiative decay channels at the surface of the flake. Secondly, we observe an exponential decrease with a fitted decay constant of -0.78 h^{-1}, which corresponds to a decrease of 45% (= $e^{-0.78}$) per hour. We assign this second phase to the layer-by-layer degradation and the formation of additional non-radiative decay channels, which is supported by a simultaneously decreasing fluorescence lifetime (Figure 2f). Finally, the intensity decays rapidly to zero which we assign to the complete degradation of the sample through the lateral degradation which is faster than the layer-by-layer degradation (we measure the fluorescence only in the center of the flake, which explains why the lateral degradation has no significant influence in the first two steps).

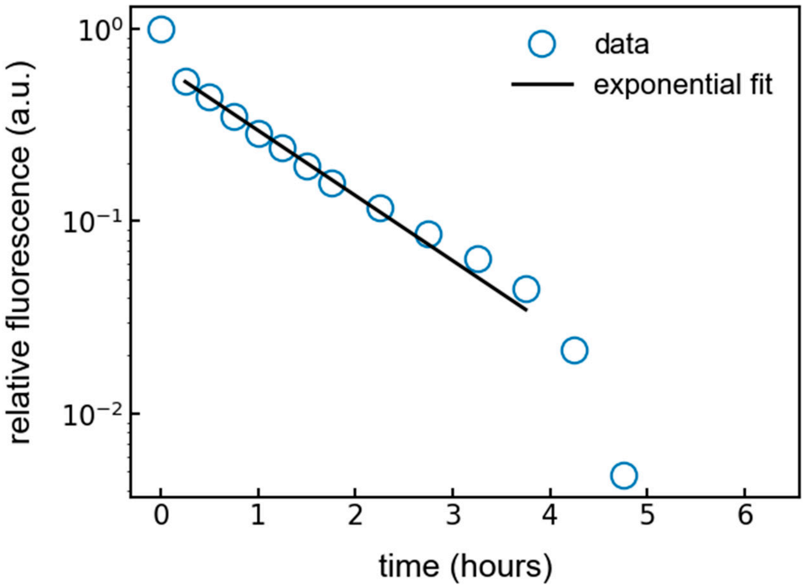

Figure S3. Total normalized (to t = 0) fluorescence intensity of an un-encapsulated perovskite flake under ambient conditions. Data shown here is the same as in Figure 2b of the main text, but with a logarithmic ordinate.

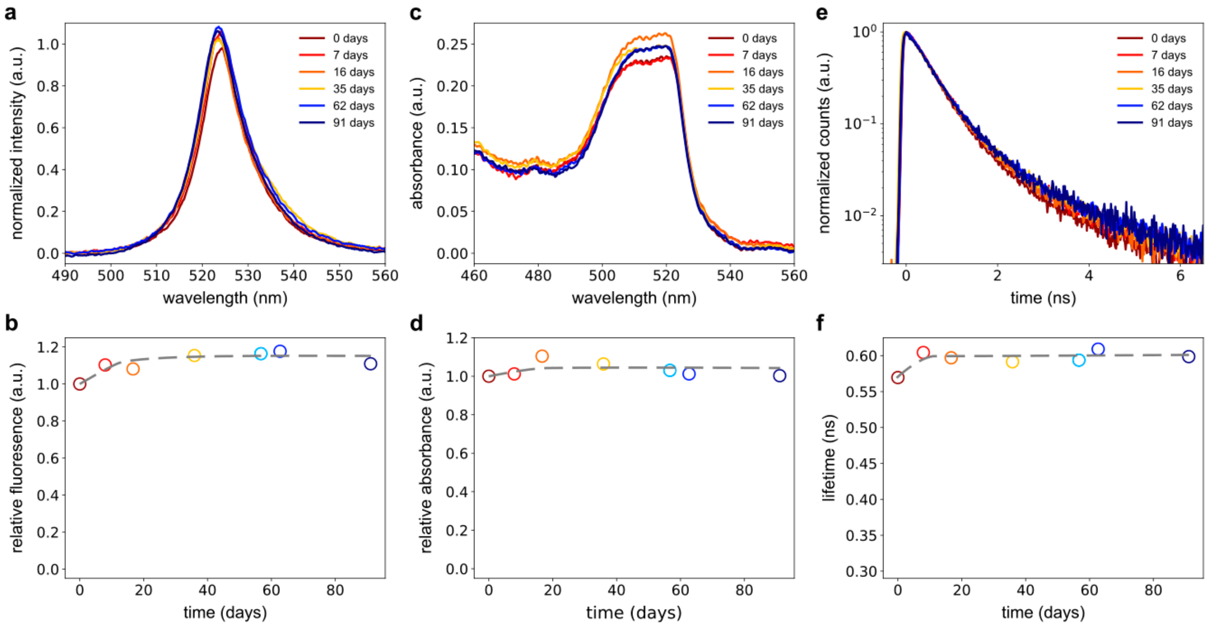

Figure S4. Same data as in Figure 3b-d, but including the traces. Spectral properties of the encapsulated PEA_2PbI_4 2D perovskite flake shown in Figure 3 of the main text for different times under ambient exposure. a, c, e) Show the fluorescence, absorbance and fluorescence lifetime traces, respectively, for different times of ambient exposure. b) Total fluorescence intensity from (a) normalized to the measured intensity at t = 0. d) Integrated absorbance (455 nm – 594 nm) from (c) and normalized to the measured absorbance at t = 0. f) Extracted 1/e lifetime from the fluorescence lifetime traces in (e). Grey lines in b), d), and f) are guides to the eye.

Figure S5 shows an encapsulated and an un-encapsulated perovskite flake for different times under ambient exposure. While the un-encapsulated flake readily degrades within one day, the hBN encapsulated flake is stable for a much longer time. However, the hBN seal of the encapsulated flake has a leak in the seal (indicated by an arrow), which allows air molecules to slowly diffuse towards the perovskite flake. Nevertheless, it is interesting to note that the center of the flake remains mainly unaffected by the degradation even after 1 month, underlining that the main purpose of the hBN seal is to shield the perovskite from air molecules.

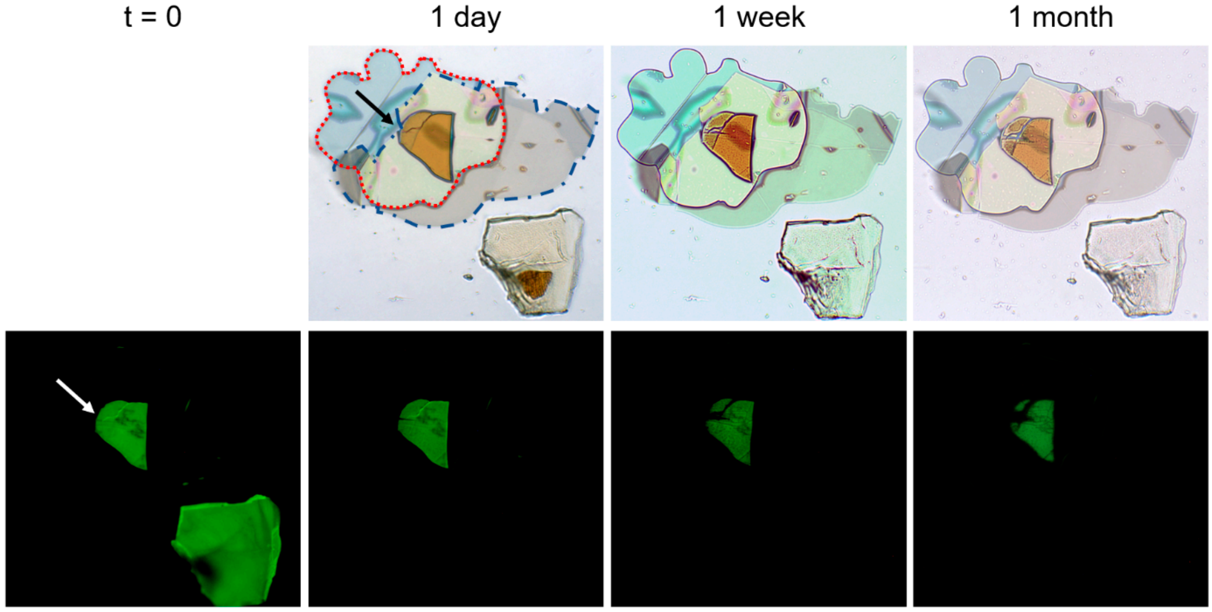

Figure S5. Transmission (top row) and fluorescence (bottom row) micrographs of an un-encapsulated (bottom right of each micrograph) and partially encapsulated (top left of each micrograph) perovskite flake for different times under ambient conditions. The four columns of the figure correspond to 0, 1

day, 1 week, and 1 month of ambient exposure. The un-encapsulated flake degrades within one day, while the partially encapsulated flake is stable for a much longer time. However, the hBN seal of the partially encapsulated flake has a leak in the seal (indicated by an arrow), which allows air molecules to slowly diffuse towards the perovskite flake leading to the degradation of the flake which spreads from the leak inwards.
